# Diagnosis and Management of Denosumab-Induced Hypocalcemia and Hypophosphatemia in the Setting of Metastatic Prostate Cancer

**DOI:** 10.7759/cureus.20928

**Published:** 2022-01-04

**Authors:** Michael G Flood, Mallory A Rowley, Alina Basnet

**Affiliations:** 1 Department of Hematology and Oncology, State University of New York Upstate Medical University, Syracuse, USA

**Keywords:** drug-related side effects and adverse reactions, electrolytes abnormalities, prostate cancer, hypocalcemia, hypophosphatemia, denosumab

## Abstract

Denosumab, a receptor activator of nuclear factor kappa-Β ligand (RANK-L) monoclonal antibody used in osteoporosis and various malignancies, has been shown to cause hypocalcemia shortly after initiation of treatment. There have been few case reports of electrolyte abnormalities in patients managed with long-term treatment with this medication. This report presents the case of a 43-year-old male with metastatic prostate cancer who presented with severe hypophosphatemia and hypocalcemia, initially resistant to repletion. After more aggressive and persistent repletion with IV calcium, phosphorus, and vitamin D, as well as time for the denosumab to dissipate, the patient’s electrolytes stabilized and he was able to be discharged with oral replacement and close follow-up. Therefore, long-term monitoring of electrolytes for patients on denosumab should be carefully considered.

## Introduction

Calcium and phosphorus serum homeostasis is maintained in part via regulation by parathyroid hormone (PTH), which acts at the level of the bones, kidneys, and small intestine. PTH prevents the reabsorption of phosphorus and stimulates vitamin D production in the proximal tubules, decreasing calcium excretion in the distal tubules. It then stimulates receptor activator of nuclear factor kappa-Β ligand (RANK-L) effects at the level of osteoblasts to increase calcium and phosphorus efflux from bone [1,2]. When calcium levels drop in the bloodstream, the feedback loop via the parathyroid glands located posterior to the thyroid is activated resulting in secretion of PTH. Fibroblast growth factor 23 (FGF23), another hormonal mediator, also participates in phosphorus regulation in order to match bone mineralization, counteracts the effects of vitamin D, and plays a key role in malignancy-induced hypophosphatemia [3,4].

Given that bone metastases occur in approximately 75% of metastatic cancer, activation of osteoclasts causes significant bone resorption which contributes to pathological fractures and other skeletal sequelae [5]. Denosumab is a human monoclonal antibody that blocks receptor activator RANK-L thereby preventing osteoclast differentiation and decreasing bone resorption [6]. This results in a bone-specific PTH resistance that causes increased phosphorus renal excretion even without the efflux of phosphorus from the bone, thereby resulting in hypophosphatemia [7]. In addition to its therapeutic use in osteoporosis, denosumab is also utilized for humoral hypercalcemia in the setting of metastatic cancer. In both patient populations, it has contributed to cases of severe hypocalcemia and hypophosphatemia. While some patients experience severe weakness and tetany, even those with asymptomatic electrolyte abnormalities can display electrocardiogram changes that predispose them to life-threatening cardiac arrhythmias [8].

Most cases of electrolyte abnormalities following denosumab administration cite serum calcium and phosphate levels rapidly decreasing within one to two weeks after administration and recovering within four weeks [9]. Recovery was achieved with replacement using intravenous calcium gluconate and oral supplementation with calcium, ergocalciferol, calcitriol, and oral phosphate if warranted [6]. Case presentations in the literature additionally cite low body mass index, glomerular filtration rate, menopause, bone and prostate malignancies, and vitamin D deficiencies as risk factors that predispose patients to adverse electrolyte imbalances. While a 2016 study demonstrated that a quarter of patients who receive denosumab develop persistent asymptomatic hypocalcemia, [8] we present the case of a patient with a history of prostate cancer who developed refractory symptomatic severe hypocalcemia, hypophosphatemia, and metabolic acidosis following denosumab.

## Case presentation

A 43-year-old male with a past medical history of prostate cancer with metastasis to the brain, bone, and retroperitoneal lymph nodes, presented with neurological complaints of dizziness and double vision and was admitted to the inpatient oncology service. Upon further evaluation with MRI, these symptoms were deemed to be secondary to the progression of disease in his brain. He had been treated in the past with various regimens which included, at various times, leuprolide, bicalutamide, enzalutamide, docetaxel, carboplatin, cabazitaxel, pegfilgrastim, denosumab, nivolumab, and ipilimumab, as well as radiation therapy. He underwent radiation to the brain lesion which resolved his neurological issues; however, during his hospital course, he was found to have several severe electrolyte abnormalities, including persistent hypocalcemia and hypophosphatemia. The patient's electrolytes on admission, post-treatment, and reference ranges are presented in Table 1. On admission, prior to MRI and radiation, his total calcium was decreased with ionized calcium markedly decreased as well, and phosphorus was extremely low. Also, his alkaline phosphatase was markedly elevated. Moreover, he had a concomitant metabolic acidosis with a normal anion gap due to distal (type I) renal tubular acidosis (RTA). Oral and subsequently intravenous calcium and phosphorus supplementation was given to replete the electrolytes, however, the calcium and phosphorus remained persistently low. 25-OH vitamin D level was on the very low end of the reference range. The differential diagnosis at this point was rather broad, as it included Fanconi syndrome, medication side effects, tumor-induced osteomalacia, and tumor PTH-related protein (PTHrp) secretion, among others. Further workup included urine electrolytes, calcitriol, PTH, PTHrp, uric acid, and FGF23. Urine electrolytes demonstrated significant phosphaturia, with urine phosphorus increased. Urine calcium and uric acid, as well as other electrolytes, were all within range. PTH was elevated and PTHrp was negative. Finally, FGF23, an important phosphorus regulator, was measured to be elevated.

**Table 1:** Laboratory values Various laboratory values, both pre- and post-treatment (if applicable), as well as reference ranges for our institution. PTH: parathyroid hormone; PTHrp: PTH-related protein; FGF23: fibroblast growth factor 23.

Name	Initial value	Post-treatment	Reference Range
Total Calcium	7.0	8.0-8.6	8.6-10.3 mg/dL
Ionized Calcium	0.91	1.1-1.2	1.13-1.32 mg/dL
Phosphorus	0.4	2.2-2.7	2.5-4.5 mg/dL
Alkaline phosphatase	905	n/a	40-129 U/L
Phosphate (urine)	1606	n/a	400-1300 mg/24 hr
25-Vitamin D	33	n/a	>30 ng/mL
PTH (intact)	332	n/a	15-65 pg/mL
PTHrp	negative	n/a	+/-
FGF23	240	n/a	44-215 RU/mL

An even more aggressive electrolyte repletion strategy was adopted, based upon the probable toxicity from denosumab given the decreased calcium, phosphorus, activated vitamin D, and lack of other positive findings suggesting other diagnoses. He had been administered his last dose of denosumab (Xgeva) two and a half weeks prior to admission. This included IV sodium phosphate, oral potassium phosphate, IV calcium gluconate, oral calcium citrate, calcitriol, cholecalciferol, and sodium bicarbonate for the RTA. We also administered the calcium and phosphorus separately to avoid calcium interference with phosphorus absorption. Over time, his total calcium, ionized calcium, and phosphorus had recovered to reference ranges. Given this marked clinical improvement and stability, the patient was ultimately discharged and advised to follow up consistently with nephrology for chronic management of oral electrolyte replacement.

## Discussion

This patient provided a complex diagnostic challenge that has not been specifically reported in the literature previously. Despite an initially broad differential diagnosis, the laboratory results obtained following the nephrology consult aided in narrowing the differential. Fanconi syndrome was ruled out as a proximal tubulopathy as seen in this disease that would cause universal solute wasting rather than a phosphorus-specific pathology. Also, as PTHrp came back negative, its involvement was eliminated. At this point, tumor-induced osteomalacia and an adverse effect from medications were the remaining differential diagnoses. FGF23, a marker for tumor-induced osteomalacia, was slightly above normal; however, in many cases of this condition, this level is drastically elevated [10]. Also, PTH was drastically elevated in this patient, while FGF23 has been shown to suppress PTH secretion, thereby reducing the likelihood of FGF23 being the main cause in this case [11]. Moreover, while still possible given slightly elevated FGF23 levels, tumor-induced osteomalacia would likely not have improved without successful treatment of the inciting prostate cancer. Therefore, we more seriously considered his medications as a potential cause. As discussed in the introduction, hypocalcemia and hypophosphatemia have been commonly reported effects when starting this medication, however, there are limited reports of electrolyte abnormalities arising after extended periods of time on the drug. However, given the lack of response in the patient’s lab values over a period of two weeks, we determined that denosumab was the most likely culprit, and decided to pursue even more aggressive repletion strategies. The patient’s improvement began about one month after his last administration of denosumab; the half-life of denosumab is approximately 32 days, further suggesting the drug’s involvement [12]. With the likely culprit identified, the patient was ultimately discharged with electrolyte supplementation, oncology and nephrology follow-up, and a recommendation to avoid further treatment with denosumab. He has since had consistent follow-up and his electrolytes are now stable.

The mechanism of this patient’s electrolyte abnormalities is unproven, however, it most likely involves the calcium-parathyroid-vitamin D axis. Denosumab acts as a RANK-L ligand antibody, preventing osteoclast activation and the resulting bone resorption [13]. As a result, calcium remained bound in the patient’s bones rather than in the serum, causing the parathyroid gland to respond to this perceived hypocalcemia by secreting abnormally high levels of PTH [14]. PTH normally results in increased calcium reabsorption and phosphorus excretion by the kidneys; however, this patient also had a vitamin D deficiency, potentially secondary to elevated FGF23 and resulting inhibition of 1-alpha hydroxylase which typically activates inactive 25-OH vitamin D into active 1,25-OH vitamin D. As a result of this diminished vitamin D and decreased bone resorption, the patient was excreting phosphorus more than usual and was unable to maintain normal levels of calcium or phosphorus [15]. This mechanism explains why repletion was initially unsuccessful as the denosumab continued to exert its effect on bone. However, as the denosumab was slowly eliminated, calcium was able to leave the bone, resulting in decreased PTH. Moreover, vitamin D repletion assisted in the reabsorption of both calcium and phosphorus by the kidneys. Therefore, the patient was successfully able to maintain the electrolyte levels with adequate repletion.

## Conclusions

Based on the findings in this patient and other case reports of electrolyte abnormalities in patients taking denosumab, we recommend more consistent, long-term monitoring of calcium, phosphorus, vitamin D, and PTH in patients taking denosumab. While these electrolyte abnormalities have been studied extensively in the short-term period, these complications have been rarely noted in patients receiving ongoing treatment with denosumab long term. This case report will aid in detecting severe electrolyte abnormalities in patients before serious repercussions can occur. At times, such electrolyte abnormalities can take weeks to months to normalize even with aggressive replacements.
